# Bis(2,4-dimeth­oxy­phen­yl)(phen­yl)phosphine selenide

**DOI:** 10.1107/S1600536810049366

**Published:** 2010-12-11

**Authors:** Alfred Muller

**Affiliations:** a Research Centre in Synthesis and Catalysis, Department of Chemistry, University of Johannesburg (APK Campus), PO Box 524, Auckland Park, Johannesburg 2006, South Africa

## Abstract

In the title mol­ecule, C_22_H_23_O_4_PSe, the P atom has a distorted tetra­hedral environment formed by the selenide atom [P=Se = 2.1219 (5) Å] and three aryl rings. The orientations of the meth­oxy groups in the two 2,4-dimeth­oxy­phenyl ligands are distinct, as seen from the torsion angles: C—C—O—C = 14.7 (3) and 175.97 (17)° in one ligand, and −9.1 (2) and 5.1 (3)° in the other. In the crystal, weak inter­molecular C—H⋯Se inter­actions link the mol­ecules into zigzag chains propagated in [010].

## Related literature

For background to studies aimed at understanding the trans­ition metal–phosphorus bond, see: Muller *et al.* (2006 ▶); Roodt *et al.* (2003 ▶) Tolman (1977 ▶). As part of this systematic investigation we are now also studying selenium-bonded phosphorus ligands, see: Muller *et al.* (2008 ▶). For the synthesis of *ortho-*substituted aryl­alkyl­phosphanes, see: Riihimäki *et al.* (2003 ▶). For a description of the Cambridge Structural Database, see: Allen (2002 ▶).
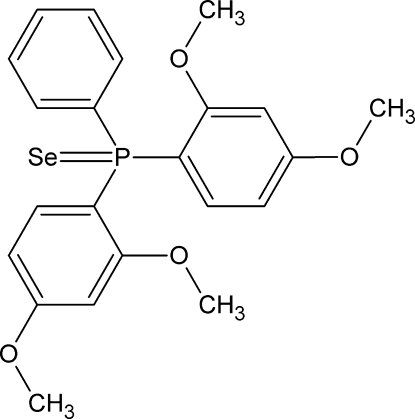


## Experimental

### 

#### Crystal data

C_22_H_23_O_4_PSe
*M* *_r_* = 461.33
Monoclinic, 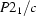

*a* = 9.3840 (13) Å
*b* = 13.3023 (14) Å
*c* = 16.667 (2) Å
β = 95.311 (4)°
*V* = 2071.6 (4) Å^3^
*Z* = 4
Mo *K*α radiation
μ = 1.92 mm^−1^
*T* = 150 K
0.34 × 0.28 × 0.06 mm

#### Data collection

Bruker X8 APEXII 4K Kappa CCD diffractometer
Absorption correction: multi-scan (*SADABS*; Bruker, 2004 ▶) *T*_min_ = 0.566, *T*_max_ = 0.891
24588 measured reflections
5151 independent reflections
4413 reflections with *I* > 2σ(*I*)
*R*_int_ = 0.030

#### Refinement

*R*[*F* ^2^ > 2σ(*F* ^2^)] = 0.029
*wR*(*F* ^2^) = 0.078
*S* = 1.04
5151 reflections
257 parameters
H-atom parameters constrained
Δρ_max_ = 1.41 e Å^−3^
Δρ_min_ = −0.20 e Å^−3^

### 

Data collection: *APEX2* (Bruker, 2005 ▶); cell refinement: *SAINT-Plus* (Bruker, 2004 ▶); data reduction: *SAINT-Plus* and *XPREP* (Bruker, 2004 ▶); program(s) used to solve structure: *SIR97* (Altomare *et al.*, 1999 ▶); program(s) used to refine structure: *SHELXL97* (Sheldrick, 2008 ▶); molecular graphics: *DIAMOND* (Brandenburg & Putz, 2005 ▶); software used to prepare material for publication: *WinGX* (Farrugia, 1999 ▶).

## Supplementary Material

Crystal structure: contains datablocks global, I. DOI: 10.1107/S1600536810049366/cv5007sup1.cif

Structure factors: contains datablocks I. DOI: 10.1107/S1600536810049366/cv5007Isup2.hkl

Additional supplementary materials:  crystallographic information; 3D view; checkCIF report

## Figures and Tables

**Table 1:** Hydrogen-bond geometry (Å, °)

*D*—H⋯*A*	*D*—H	H⋯*A*	*D*⋯*A*	*D*—H⋯*A*
C3—H3*C*⋯Se^i^	0.98	2.94	3.8774 (19)	160

Symmetry code: (i) 
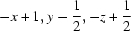
.

**Fig. 1.**
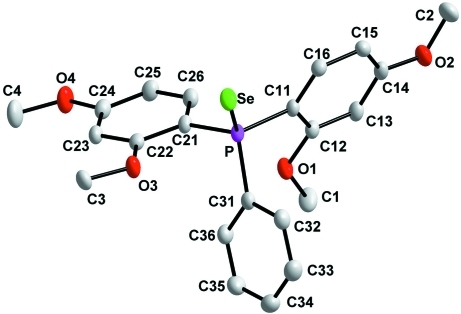
View of (I) (50% probability displacement ellipsoids). H atoms have been omitted for clarity. For the C atoms, the first digit indicates the ring number and the second digit indicates the position of the atom in the ring.

## supplementary crystallographic information

### Comment

There has been extensive development in understanding the transition metal phosphorous bond by various groups, including our own, with various techniques such as single-crystal X-ray crystallography, multi nuclear NMR and IR (Roodt *et al.*, 2003). As part of this systematic investigation we are now also studying selenium bonded phosphorus ligands (see Muller *et al.*, 2008) This way there is no steric crowding effect, albeit crystal packing effects, as normally found in transition metal complexes with bulky ligands, *e.g.* in *trans*-[Rh(CO)Cl{P(OC_6_H_5_)_3_}_2_] cone angles variation from 156° to 167° was observed for the two phosphite ligands (Muller *et al.*, 2006). The *J*(^31^P-^77^Se) coupling can also be used as an additional probe to obtain more information regarding the nature of the phosphorous bond. Reported here is the first single-crystal structure of the compound PPh(2,4-OMe-C_6_H_3_)_2_ to date (Cambridge Structural Database; Version 5.31, update of August; Allen, 2002).

Crystals of the title compound, (I), packs in the *P*2_1_/*c* (*Z*=4) space group with the molecules lying on general positions. All geometrical features of the molecule (Allen, 2002) are as expected with the selenium atom and the three aryl groups adopting a distorted arrangement about phosphorous (see Fig. 1 and Table 1). The cone angle was found to be 176.9° when the Se—P distance is adjusted to 2.28 Å (the default value used in Tolman, 1977) Two different orientations for the methoxy moieties are noted and is probably due to some weak interactions (Table 1) forcing it into the conformations observed.

### Experimental

PPh(2,4-OMe-C_6_H_3_)_2_ were prepared either by direct *ortho* metallation of anisole with BuLi followed the addition of the appropriate chlorophosphine or by metal/halogen exchange between BuLi and 1-bromo-2,4-dimethoxybenzene followed by the addition of PPhCl_2_ according to established methods (Riihimäki *et al.* 2003).

Eqimolar amounts of KSeCN and the PPh(2,4-OMe-C_6_H_3_)_2_ compound (*ca* 0.04 mmol) were dissolved in the minimum amounts of methanol (10 – 20 ml). The KSeCN solution was added dropwise (5 min.) to the phosphine solution with stirring at room temperature. The final solution was left to evaporate slowly until dry to give crystals suitable for a single-crystal X-ray study.

Analytical data: ^31^P {H} NMR (CDCl_3_, 121.42 MHz): For PPh(2,4-OMe-C_6_H_3_)_2_δ = -36.54 (*s*) For SePPh(2,4-OMe-C_6_H_3_)_2_δ = 20.45 (t, ^1^J_P—Se_ = 717.5 Hz)

### Refinement

The aromatic and methylene H atoms were placed in geometrically idealized positions (C—H = 0.93 – 0.98 Å) and constrained to ride on their parent atoms with *U*_iso_(H) = 1.2*U*_eq_(C) and *U*_iso_(H) = 1.5*U*_eq_(C) respectively, with torsion angles refined from the electron density for the methyl groups. The highest residual electron density was located 0.64 Å from H25.

### Crystal data

**Table:**

C_22_H_23_O_4_PSe	*F*(000) = 944
*M**_r_* = 461.33	*D*_x_ = 1.479 Mg m^−^^3^
Monoclinic, *P*2_1_/*c*	Mo *K*α radiation, λ = 0.71073 Å
Hall symbol: -P 2ybc	Cell parameters from 5013 reflections
*a* = 9.3840 (13) Å	θ = 2.7–28.3°
*b* = 13.3023 (14) Å	µ = 1.92 mm^−^^1^
*c* = 16.667 (2) Å	*T* = 150 K
β = 95.311 (4)°	Plate, colourless
*V* = 2071.6 (4) Å^3^	0.34 × 0.28 × 0.06 mm
*Z* = 4	

### Data collection

**Table:**

Bruker X8 APEXII 4K Kappa CCD diffractometer	5151 independent reflections
graphite	4413 reflections with *I* > 2σ(*I*)
Detector resolution: 8.4 pixels mm^-1^	*R*_int_ = 0.030
ω & φ scans	θ_max_ = 28.3°, θ_min_ = 2.0°
Absorption correction: multi-scan (*SADABS*; Bruker, 2004)	*h* = −12→12
*T*_min_ = 0.566, *T*_max_ = 0.891	*k* = −17→16
24588 measured reflections	*l* = −22→22

### Refinement

**Table:**

Refinement on *F*^2^	0 restraints
Least-squares matrix: full	H-atom parameters constrained
*R*[*F*^2^ > 2σ(*F*^2^)] = 0.029	*w* = 1/[σ^2^(*F*_o_^2^) + (0.0398*P*)^2^ + 1.21*P*] where *P* = (*F*_o_^2^ + 2*F*_c_^2^)/3
*wR*(*F*^2^) = 0.078	(Δ/σ)_max_ = 0.001
*S* = 1.04	Δρ_max_ = 1.41 e Å^−^^3^
5151 reflections	Δρ_min_ = −0.20 e Å^−^^3^
257 parameters	

### Special details

**Table:**

Experimental. The intensity data was collected on a Bruker X8 Apex II 4 K Kappa CCD diffractometer using an exposure time of 10 s/frame. A total of 1125 frames were collected with a frame width of 0.5° covering up to θ = 28.25° with 99.7% completeness accomplished.
Geometry. All e.s.d.'s (except the e.s.d. in the dihedral angle between two l.s. planes) are estimated using the full covariance matrix. The cell e.s.d.'s are taken into account individually in the estimation of e.s.d.'s in distances, angles and torsion angles; correlations between e.s.d.'s in cell parameters are only used when they are defined by crystal symmetry. An approximate (isotropic) treatment of cell e.s.d.'s is used for estimating e.s.d.'s involving l.s. planes.

### Fractional atomic coordinates and isotropic or equivalent isotropic displacement parameters (Å^2^)

**Table:**

	*x*	*y*	*z*	*U*_iso_*/*U*_eq_	
Se	0.31675 (2)	0.914803 (13)	0.136815 (10)	0.02690 (7)	
P	0.33832 (5)	0.78342 (3)	0.06676 (2)	0.01886 (10)	
C11	0.22855 (19)	0.79244 (13)	−0.02854 (9)	0.0193 (3)	
C12	0.24045 (19)	0.72308 (12)	−0.09174 (10)	0.0207 (3)	
C13	0.1607 (2)	0.73473 (14)	−0.16557 (10)	0.0234 (4)	
H13	0.1699	0.6879	−0.2078	0.028*	
C14	0.06675 (19)	0.81588 (14)	−0.17707 (10)	0.0229 (3)	
C15	0.05290 (19)	0.88571 (14)	−0.11587 (11)	0.0234 (3)	
H15	−0.0107	0.9411	−0.1241	0.028*	
C16	0.13423 (19)	0.87254 (13)	−0.04265 (10)	0.0221 (3)	
H16	0.1251	0.9199	−0.0007	0.027*	
C1	0.3774 (3)	0.58741 (14)	−0.13985 (12)	0.0330 (5)	
H1A	0.2998	0.5417	−0.1591	0.049*	
H1B	0.4627	0.5483	−0.1213	0.049*	
H1C	0.3996	0.632	−0.1838	0.049*	
O1	0.33407 (15)	0.64596 (9)	−0.07480 (7)	0.0271 (3)	
C2	−0.1108 (2)	0.89791 (18)	−0.26447 (13)	0.0367 (5)	
H2A	−0.1793	0.8935	−0.2237	0.055*	
H2B	−0.1616	0.89	−0.3182	0.055*	
H2C	−0.0633	0.9636	−0.2607	0.055*	
O2	−0.00636 (14)	0.82027 (11)	−0.25114 (8)	0.0297 (3)	
C21	0.28849 (19)	0.66839 (13)	0.11441 (10)	0.0206 (3)	
C22	0.35987 (19)	0.63756 (13)	0.18822 (10)	0.0210 (3)	
C23	0.3213 (2)	0.54914 (13)	0.22579 (10)	0.0229 (3)	
H23	0.371	0.5286	0.2753	0.028*	
C24	0.2101 (2)	0.49165 (13)	0.19012 (11)	0.0254 (4)	
C25	0.1364 (2)	0.52101 (14)	0.11711 (11)	0.0265 (4)	
H25	0.0601	0.4812	0.0929	0.032*	
C26	0.1761 (2)	0.60867 (14)	0.08048 (10)	0.0241 (4)	
H26	0.1257	0.6288	0.031	0.029*	
C3	0.5321 (2)	0.67676 (15)	0.29847 (11)	0.0275 (4)	
H3A	0.4586	0.6798	0.3365	0.041*	
H3B	0.6066	0.7267	0.3136	0.041*	
H3C	0.5748	0.6095	0.2998	0.041*	
O3	0.46854 (14)	0.69765 (10)	0.21903 (7)	0.0273 (3)	
C4	0.2265 (3)	0.37696 (16)	0.30097 (12)	0.0376 (5)	
H4A	0.3269	0.3586	0.2978	0.056*	
H4B	0.1746	0.3194	0.3206	0.056*	
H4C	0.2209	0.4336	0.3381	0.056*	
O4	0.16333 (17)	0.40516 (10)	0.22236 (9)	0.0352 (3)	
C31	0.52039 (19)	0.76605 (13)	0.04006 (10)	0.0206 (3)	
C32	0.5942 (2)	0.84791 (14)	0.01235 (11)	0.0258 (4)	
H32	0.5517	0.9128	0.011	0.031*	
C33	0.7297 (2)	0.83512 (16)	−0.01327 (12)	0.0315 (4)	
H33	0.7795	0.8912	−0.0323	0.038*	
C35	0.7206 (2)	0.65944 (16)	0.01708 (12)	0.0321 (4)	
H35	0.7641	0.5949	0.0192	0.039*	
C36	0.5850 (2)	0.67188 (14)	0.04235 (11)	0.0276 (4)	
H36	0.5358	0.6156	0.0614	0.033*	
C34	0.7925 (2)	0.74100 (17)	−0.01121 (12)	0.0336 (4)	
H343	0.8849	0.7324	−0.0292	0.04*	

### Atomic displacement parameters (Å^2^)

**Table:**

	*U*^11^	*U*^22^	*U*^33^	*U*^12^	*U*^13^	*U*^23^
Se	0.04157 (12)	0.02049 (10)	0.01806 (10)	0.00414 (7)	−0.00027 (7)	−0.00378 (6)
P	0.0259 (2)	0.0170 (2)	0.01349 (19)	0.00107 (16)	0.00077 (16)	0.00097 (15)
C11	0.0237 (8)	0.0208 (8)	0.0134 (7)	−0.0003 (6)	0.0014 (6)	0.0018 (6)
C12	0.0262 (9)	0.0186 (8)	0.0174 (8)	−0.0006 (6)	0.0025 (6)	0.0025 (6)
C13	0.0297 (9)	0.0244 (9)	0.0158 (7)	−0.0029 (7)	0.0005 (7)	0.0001 (6)
C14	0.0217 (8)	0.0286 (9)	0.0180 (8)	−0.0046 (7)	−0.0011 (6)	0.0050 (7)
C15	0.0213 (8)	0.0263 (9)	0.0227 (8)	0.0026 (7)	0.0020 (7)	0.0049 (7)
C16	0.0248 (9)	0.0235 (9)	0.0184 (8)	0.0007 (7)	0.0039 (6)	0.0006 (6)
C1	0.0545 (13)	0.0214 (9)	0.0234 (9)	0.0075 (8)	0.0059 (9)	−0.0047 (7)
O1	0.0419 (8)	0.0225 (6)	0.0164 (6)	0.0092 (6)	0.0000 (5)	−0.0015 (5)
C2	0.0274 (10)	0.0493 (13)	0.0317 (10)	0.0067 (9)	−0.0061 (8)	0.0054 (9)
O2	0.0304 (7)	0.0358 (7)	0.0214 (6)	0.0008 (6)	−0.0065 (5)	0.0035 (6)
C21	0.0286 (9)	0.0181 (8)	0.0155 (7)	0.0013 (7)	0.0039 (6)	0.0019 (6)
C22	0.0247 (9)	0.0206 (8)	0.0179 (8)	0.0020 (7)	0.0041 (6)	0.0008 (6)
C23	0.0299 (9)	0.0210 (8)	0.0182 (8)	0.0028 (7)	0.0039 (7)	0.0043 (6)
C24	0.0340 (10)	0.0195 (8)	0.0234 (8)	0.0001 (7)	0.0064 (7)	0.0022 (7)
C25	0.0333 (10)	0.0251 (9)	0.0212 (8)	−0.0053 (7)	0.0027 (7)	−0.0013 (7)
C26	0.0309 (9)	0.0234 (9)	0.0179 (8)	0.0012 (7)	0.0019 (7)	0.0004 (6)
C3	0.0284 (9)	0.0308 (10)	0.0221 (8)	0.0028 (8)	−0.0045 (7)	0.0052 (7)
O3	0.0330 (7)	0.0272 (7)	0.0205 (6)	−0.0056 (5)	−0.0038 (5)	0.0062 (5)
C4	0.0573 (14)	0.0247 (10)	0.0301 (10)	−0.0049 (9)	−0.0003 (9)	0.0107 (8)
O4	0.0496 (9)	0.0254 (7)	0.0299 (7)	−0.0115 (6)	−0.0010 (6)	0.0090 (5)
C31	0.0246 (8)	0.0217 (8)	0.0152 (7)	0.0004 (6)	0.0000 (6)	0.0013 (6)
C32	0.0313 (10)	0.0216 (9)	0.0241 (9)	−0.0002 (7)	0.0003 (7)	0.0035 (7)
C33	0.0316 (10)	0.0324 (10)	0.0303 (10)	−0.0052 (8)	0.0028 (8)	0.0055 (8)
C35	0.0351 (11)	0.0305 (10)	0.0313 (10)	0.0093 (8)	0.0060 (8)	0.0051 (8)
C36	0.0335 (10)	0.0238 (9)	0.0260 (9)	0.0034 (7)	0.0059 (7)	0.0068 (7)
C34	0.0282 (10)	0.0425 (12)	0.0308 (10)	0.0039 (9)	0.0065 (8)	0.0052 (9)

### Geometric parameters (Å, °)

**Table:**

Se—P	2.1219 (5)	C23—C24	1.383 (3)
P—C21	1.8054 (17)	C23—H23	0.95
P—C11	1.8154 (17)	C24—O4	1.359 (2)
P—C31	1.8196 (18)	C24—C25	1.398 (3)
C11—C16	1.391 (2)	C25—C26	1.383 (3)
C11—C12	1.412 (2)	C25—H25	0.95
C12—O1	1.363 (2)	C26—H26	0.95
C12—C13	1.389 (2)	C3—O3	1.428 (2)
C13—C14	1.395 (3)	C3—H3A	0.98
C13—H13	0.95	C3—H3B	0.98
C14—O2	1.357 (2)	C3—H3C	0.98
C14—C15	1.395 (3)	C4—O4	1.437 (2)
C15—C16	1.389 (2)	C4—H4A	0.98
C15—H15	0.95	C4—H4B	0.98
C16—H16	0.95	C4—H4C	0.98
C1—O1	1.425 (2)	C31—C36	1.391 (3)
C1—H1A	0.98	C31—C32	1.392 (2)
C1—H1B	0.98	C32—C33	1.389 (3)
C1—H1C	0.98	C32—H32	0.95
C2—O2	1.427 (2)	C33—C34	1.383 (3)
C2—H2A	0.98	C33—H33	0.95
C2—H2B	0.98	C35—C34	1.383 (3)
C2—H2C	0.98	C35—C36	1.387 (3)
C21—C26	1.397 (3)	C35—H35	0.95
C21—C22	1.406 (2)	C36—H36	0.95
C22—O3	1.359 (2)	C34—H343	0.95
C22—C23	1.396 (2)		
			
C21—P—C11	106.96 (8)	C24—C23—C22	119.33 (16)
C21—P—C31	106.69 (8)	C24—C23—H23	120.3
C11—P—C31	105.31 (8)	C22—C23—H23	120.3
C21—P—Se	114.47 (6)	O4—C24—C23	123.80 (17)
C11—P—Se	110.59 (6)	O4—C24—C25	115.40 (17)
C31—P—Se	112.25 (6)	C23—C24—C25	120.80 (16)
C16—C11—C12	117.86 (15)	C26—C25—C24	119.22 (17)
C16—C11—P	119.94 (13)	C26—C25—H25	120.4
C12—C11—P	122.12 (13)	C24—C25—H25	120.4
O1—C12—C13	123.50 (15)	C25—C26—C21	121.63 (16)
O1—C12—C11	115.53 (15)	C25—C26—H26	119.2
C13—C12—C11	120.97 (16)	C21—C26—H26	119.2
C12—C13—C14	119.33 (16)	O3—C3—H3A	109.5
C12—C13—H13	120.3	O3—C3—H3B	109.5
C14—C13—H13	120.3	H3A—C3—H3B	109.5
O2—C14—C15	124.28 (17)	O3—C3—H3C	109.5
O2—C14—C13	114.71 (16)	H3A—C3—H3C	109.5
C15—C14—C13	121.01 (16)	H3B—C3—H3C	109.5
C16—C15—C14	118.54 (17)	C22—O3—C3	118.07 (14)
C16—C15—H15	120.7	O4—C4—H4A	109.5
C14—C15—H15	120.7	O4—C4—H4B	109.5
C15—C16—C11	122.29 (16)	H4A—C4—H4B	109.5
C15—C16—H16	118.9	O4—C4—H4C	109.5
C11—C16—H16	118.9	H4A—C4—H4C	109.5
O1—C1—H1A	109.5	H4B—C4—H4C	109.5
O1—C1—H1B	109.5	C24—O4—C4	117.42 (16)
H1A—C1—H1B	109.5	C36—C31—C32	118.98 (17)
O1—C1—H1C	109.5	C36—C31—P	121.64 (14)
H1A—C1—H1C	109.5	C32—C31—P	119.29 (14)
H1B—C1—H1C	109.5	C33—C32—C31	120.27 (18)
C12—O1—C1	118.54 (14)	C33—C32—H32	119.9
O2—C2—H2A	109.5	C31—C32—H32	119.9
O2—C2—H2B	109.5	C34—C33—C32	120.24 (18)
H2A—C2—H2B	109.5	C34—C33—H33	119.9
O2—C2—H2C	109.5	C32—C33—H33	119.9
H2A—C2—H2C	109.5	C34—C35—C36	120.01 (19)
H2B—C2—H2C	109.5	C34—C35—H35	120
C14—O2—C2	117.07 (15)	C36—C35—H35	120
C26—C21—C22	117.95 (15)	C35—C36—C31	120.61 (18)
C26—C21—P	121.36 (13)	C35—C36—H36	119.7
C22—C21—P	120.67 (14)	C31—C36—H36	119.7
O3—C22—C23	122.88 (15)	C33—C34—C35	119.89 (19)
O3—C22—C21	116.05 (15)	C33—C34—H343	120.1
C23—C22—C21	121.05 (16)	C35—C34—H343	120.1
			
C21—P—C11—C16	118.16 (14)	P—C21—C22—O3	−1.7 (2)
C31—P—C11—C16	−128.58 (14)	C26—C21—C22—C23	1.1 (3)
Se—P—C11—C16	−7.10 (16)	P—C21—C22—C23	179.51 (13)
C21—P—C11—C12	−65.25 (16)	O3—C22—C23—C24	−179.45 (16)
C31—P—C11—C12	48.01 (16)	C21—C22—C23—C24	−0.7 (3)
Se—P—C11—C12	169.49 (13)	C22—C23—C24—O4	−179.15 (17)
C16—C11—C12—O1	−179.90 (15)	C22—C23—C24—C25	0.1 (3)
P—C11—C12—O1	3.4 (2)	O4—C24—C25—C26	179.37 (17)
C16—C11—C12—C13	0.1 (3)	C23—C24—C25—C26	0.0 (3)
P—C11—C12—C13	−176.53 (14)	C24—C25—C26—C21	0.4 (3)
O1—C12—C13—C14	179.58 (16)	C22—C21—C26—C25	−0.9 (3)
C11—C12—C13—C14	−0.4 (3)	P—C21—C26—C25	−179.33 (14)
C12—C13—C14—O2	−179.81 (16)	C23—C22—O3—C3	−9.1 (2)
C12—C13—C14—C15	0.6 (3)	C21—C22—O3—C3	172.14 (16)
O2—C14—C15—C16	−179.96 (16)	C23—C24—O4—C4	5.1 (3)
C13—C14—C15—C16	−0.4 (3)	C25—C24—O4—C4	−174.23 (18)
C14—C15—C16—C11	0.1 (3)	C21—P—C31—C36	12.52 (17)
C12—C11—C16—C15	0.1 (3)	C11—P—C31—C36	−100.93 (16)
P—C11—C16—C15	176.81 (14)	Se—P—C31—C36	138.67 (14)
C13—C12—O1—C1	14.7 (3)	C21—P—C31—C32	−171.12 (14)
C11—C12—O1—C1	−165.27 (17)	C11—P—C31—C32	75.43 (15)
C15—C14—O2—C2	−4.4 (3)	Se—P—C31—C32	−44.96 (15)
C13—C14—O2—C2	175.97 (17)	C36—C31—C32—C33	0.6 (3)
C11—P—C21—C26	−4.58 (17)	P—C31—C32—C33	−175.85 (14)
C31—P—C21—C26	−116.89 (15)	C31—C32—C33—C34	−0.2 (3)
Se—P—C21—C26	118.29 (14)	C34—C35—C36—C31	−0.4 (3)
C11—P—C21—C22	177.05 (14)	C32—C31—C36—C35	−0.3 (3)
C31—P—C21—C22	64.74 (16)	P—C31—C36—C35	176.06 (15)
Se—P—C21—C22	−60.07 (16)	C32—C33—C34—C35	−0.5 (3)
C26—C21—C22—O3	179.91 (15)	C36—C35—C34—C33	0.8 (3)

### Hydrogen-bond geometry (Å, °)

**Table:**

*D*—H···*A*	*D*—H	H···*A*	*D*···*A*	*D*—H···*A*
C3—H3C···Se^i^	0.98	2.94	3.8774 (19)	160.

Symmetry codes: (i) −*x*+1, *y*−1/2, −*z*+1/2.
